# Description of a new soft scale insect of the genus *Pulvinaria* Targioni Tozzetti (Hemiptera, Coccoidea, Coccidae) from Bogota, Colombia

**DOI:** 10.3897/zookeys.484.9280

**Published:** 2015-03-09

**Authors:** Hirotaka Tanaka, Takumasa Kondo

**Affiliations:** 1 Tottori Prefectural Museum, 2–124 Higashi-machi, Tottori, 680–0011 Japan; 2 Corporación Colombiana de Investigación Agropecuaria (CORPOICA), Centro de Investigación Palmira, Calle 23, Carrera 37, Continuo al Penal, Palmira, Valle, Colombia

**Keywords:** Coccid, *Ficus soatensis*, soft scale insect, insect pest, urban pest

## Abstract

A new soft scale (Hemiptera: Coccoidea: Coccidae) species, *Pulvinaria caballeroramosae* Tanaka & Kondo, **sp. n.**, is described from specimens collected on twigs of *Ficus soatensis* Dugand (Moraceae) in Bogota, Colombia. The new species resembles *Pulvinaria drymiswinteri* Kondo & Gullan, described from Chile on *Drimys winteri* J.R. Forst. & G. Forst. (Winteraceae), but differs in the distribution of preopercular pores on the dorsum, the presence of dorsal tubular ducts, dorsal microducts, and reticulation on the anal plates; and in its feeding habits, i.e., *Pulvinaria caballeroramosae* feeds on the twigs whereas *Pulvinaria drymiswinteri* feeds on the leaves of its host. A key to the Colombian species of *Pulvinaria* Targioni Tozzetti is provided.

## Introduction

With the exception of Argentina (Granara de Willink 1999), Brazil (Hempel 1900), Colombia (Mosquera 1979, 1984; Kondo 2001, 2010a, b, 2011, 2013, Kondo and Hodgson 2013, Kondo and Williams 2004, Walker 1852) and Chile (Kondo and Gullan 2010), the soft scale insect (Hemiptera: Coccoidea: Coccidae) fauna of most countries in South America remains much under explored and studied. Some important earlier taxonomic works on soft scale insects of Colombia include those by Mosquera (1979, 1984) who contributed to the understanding of the genus *Ceroplastes* in that country (Kondo 2001). There are also species lists that include soft scale insects on some fruit crops in Colombia, namely avocado (Kondo et al. 2011), citrus (Kondo et al. 2012), mango (Kondo 2009, Kondo-Rodriguez 2010) and soursop (Kondo 2008). According to the scale insect database ScaleNet (Ben-Dov et al. 2014), the family Coccidae in Colombia is composed of 41 species distributed in 17 genera, of which 13 species (32%) are only known from Colombia, namely *Akermes colombiensis* Kondo & Williams, *Bombacoccus aguacatae* Kondo, *Ceroplastes boyacensis* Mosquera, *Ceroplastes cundinamarcensis* Mosquera, *Ceroplastes martinae* Mosquera, *Ceroplastes mosquerai* Ben-Dov, *Ceroplastes ocreus* Mosquera, *Ceroplastes trochezi* Mosquera, *Coccus caudatus* Walker, *Cryptostigma philwardi* Kondo, *Foldilecanium multisetosum* Kondo, *Hemilecanium guanabana* Kondo & Hodgson and *Neotoumeyella caliensis* Kondo & Williams.

A few years ago, the second author of the present paper was informed by Mrs. Andrea Amalia Ramos-Portilla of a species of *Pulvinaria* causing damage to street trees in Bogota. Outbreaks of this *Pulvinaria* species have been known for quite some time in the capital city of Colombia where it is undoubtedly considered an urban pest. Herein we describe and illustrate this undescribed pest species of *Pulvinaria* based on adult female specimens. A key to the species of Colombian *Pulvinaria* is also presented.

## Materials and methods

In the past, the genus *Pulvinaria* had been split into several genera, e.g. *Chloropulvinaria* (Borchsenius 1952), *Eupulvinaria* (Borchsenius 1953) and *Saccharipulvinaria* (Tao et al. 1983). However, these genera have been rarely accepted by current taxonomists (e.g., Williams and Watson 1990), and taxonomy of the tribe Pulvinariini (*Pulvinaria* and related genera) is in great need of further study (Tanaka 2012). We therefore treat the genus *Pulvinaria* in the broad sense here.

The scale insect samples were collected by the second author on 5 September 2014 from street trees of *Ficus soatensis* in the city of Bogota, Colombia with the help of Mrs. Andrea Amalia Ramos Portilla. The slide-mounting method followed Tanaka (2014). The morphology of the mounted specimens was examined under a phase-contrast light microscope (Olympus BH2-PH).

The description was based on multiple slide-mounted specimens. The terminology used to describe the adult female followed that of Kondo and Hodgson (2013), who avoided using the term “pregenital disc-pores” or “perivular pores” because in some soft scale species, these multilocular pores are not restricted to the pregenital or perivulvar region, and they can be present throughout the mid-areas of the venter; thus using the term “pregenital” or “perivulvar” is misleading. The term “multilocular pore” is therefore used herein for all the pores with multiple loculi, with the exception of spiracular pores.

The type specimens are deposited in the Colección Taxonómica Nacional “Luis María Murillo”, Corpoica, C.I. Tibaitatá, Mosquera, Cundinamarca, Colombia (CTNI), the Museo Entomológico Facultad de Agronomía, Universidad Nacional de Colombia, Sede Bogotá, Bogotá, Cundinamarca, Colombia (UNAB), the National Museum of Natural History Entomological Collection, Washington, D.C., U.S.A. (USNM: Coccoidea collection held at USDA, Beltsville, Maryland), and the Tottori Prefectural Museum, Tottori, Japan (TRPM).

## Taxonomy

### Genus Pulvinaria

Taxon classificationAnimaliaHemipteraCoccidae

Targioni Tozzetti, 1866: 146.

#### Type species.

*Coccus vitis* Linnaeus, 1758: 456. By original designation and monotypy.

The new species described below is a typical member of the tribe Pulvinariini and the subfamily Coccinae, based on the definition of the tribe Pulvinariini presented by Hodgson (1994). The present species keys out to the genus *Pulvinaria* in Hodgson’s keys to subfamilies, tribes and genera of Coccidae (Hodgson 1994) and fits into his *Pulvinaria*-group, in which tubular ducts are scarce or absent on the head. However, here we treat the genus in the broad sense, as explained in the Materials and methods section.

#### Key to Colombian species of the genus *Pulvinaria*

**Table:** 

1	Most marginal setae with bifid, frayed, fimbriate, or finely split apices	**2**
–	Most marginal setae with sharply or rather bluntly pointed apices	**3**
2	Ventral tubular ducts in submarginal area of head frequent and broadly distributed. Multilocular pores mainly each with 9–11 loculi. Marginal setae usually strongly fimbriate; setal collar of most setae narrower than setal tip. Spiracles of mature specimens usually surrounded by a strongly sclerotized crescentic plate	***psidii***
–	Ventral tubular ducts in submarginal area of head scarce or absent except in area near margin. Multilocular pores mainly each with 6–7 loculi. Marginal setae usually slightly to moderately fimbriate. Spiracles of mature specimens not surrounded by a strongly sclerotized crescentic plate	***urbicola***
3	Submarginal area of head and thorax with ventral tubular ducts numerous and widespread. Dorsal setae lanceolate, each seta with a marked constriction at base. Body shape usually conspicuously elongate oval	**4**
–	Submarginal (and marginal) area of head and thorax without ventral tubular ducts. Multilocular pores mainly each with five loculi. Dorsal setae spiniform, each seta without a marked constriction at base. Body shape oval rather than elongate	***caballeroramosae* sp. n.**
4	Multilocular pores absent medially on thorax. Ventral tubular ducts present medially on thorax between mesothoracic and metathoracic coxae	***iceryi***
–	Multilocular pores present medially on thorax between mesothoracic and metathoracic coxae. Ventral tubular ducts absent medially on thorax	***elongata***

#### Notes.

Morphological character states used for separating *Pulvinaria iceryi* from *Pulvinaria elongata* were taken from Mamet (1958). Character states of *Pulvinaria urbicola* and *Pulvinaria psidii* were taken from Williams and Watson (1990) and based also on the first author’s personal observations of slide-mounted specimens collected in Japan.

### Pulvinaria caballeroramosae

Taxon classificationAnimaliaHemipteraCoccidae

Tanaka & Kondo sp. n.

http://zoobank.org/BF0B0A32-D4E2-4952-8DD9-0A8C9569B774

Figures 1 , 2

#### Proposed common names.

Spanish: Escama blanda algodonosa del caucho sabanero; English: Sabanero fig cottony scale.

#### Type series.

Holotype, adult female. Colombia, Cundinamarca, Bogotá, D.C. Barrio Salitre, Carrera 68B, con Av. La Esperanza, Esquina Noroccidental, 05.xi.2014, coll. T. Kondo & Andrea Amalia Ramos Portilla, ex branches of *Ficus soatensis* Dugand (Moraceae), 1♀ (UNAB). Paratypes, same data as holotype, 11 ♀♀ (3 at UNAB, 3 CTNI, 3 USNM and 2 at TRPM).

#### Unmounted material

**Figure 1.**
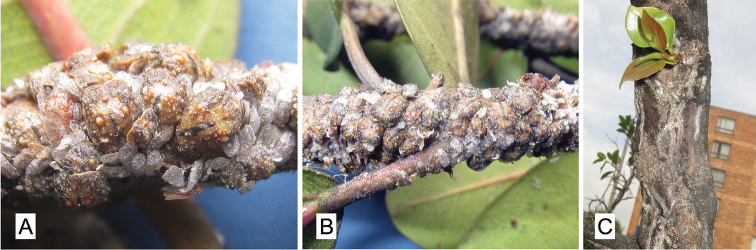
*Pulvinaria caballeroramosae* Tanaka & Kondo, sp. n.: **A** Adult females, male puparia and an alate adult male (on lower surface of twig) **B** Infestation on twig **C** Conspicuous long ovisacs on trunk of a young tree, *Ficus soatensis*. Bogota, Colombia.

(Figure 1A, B, C). Adult female in life oval, convex, 2.2–4.5 mm long, 1.9–3.8 mm wide, 0.9–2.0 mm tall, covered by a thin layer of glassy wax (Figure 1A). Body greenish brown to yellowish brown, especially around body margin, mid dorsum lighter in color, yellowish to ochre, usually with a dark mid-dorsal longitudinal line from head margin to just anterior to anal plates (Figure 1A, B). Anal plates conspicuous, reddish brown; area around anal plates generally smooth and yellowish (Figure 1A, B). Dorsal derm warty in appearance (except around anal plates), with round yellowish tubercles, especially on mid dorsum, tubercles fewer and smaller around margins and submargins; often with a pair of particularly large (two or more times wider than the anal plates) round submedial tubercles on mid dorsum, located diagonally from anal plates (Figure 1A, B). Ovisac long, four or more times the length of the adult female, produced in a straight or curved line, strongly adhered to substrate, eggs generally exposed and clearly visible through the fibrous ovisac; eggs orange, purplish or ochre in color (Figure 1C).

#### Slide-mounted specimens

(n=12). Body oval, 2.5–4.6 mm long, 2.1–3.5 mm wide, margin with very shallow indentation at each stigmatic cleft; anal cleft about 1/5–1/8 body length.

Dorsum: Derm membranous, dermal areolation not developed. Dorsal setae spiniform, frequent, scattered over entire dorsum, each 9–15 µm long with well-developed basal socket. Preopercular pores circular, each diameter 3–7 µm, rather well-sclerotized and convex, extending broadly on medial area from area just anterior to anal plates forward to about mesothorax, but usually scarce anteriorly. Only a few tubular ducts present, situated anterior to anal plates, where they are intermixed with preopercular pores; sometimes ducts also present marginally on head and thorax. Dorsal microducts frequent throughout. Simple pores present, mostly distributed evenly. Dorsal tubercles absent. Anal plates together quadrate; each plate with posterior margin slightly convex and anterior margin slightly concave, with 3–4 (usually 3) fine apical setae; each plate 223–258 µm long, 128–166 µm wide, with supporting bar and reticulation on area near lateral angle. Ano-genital fold with four or five pairs of setae along anterior margin and one to three pairs laterally. Anal ring bearing about 10–12 setae. Eyespots present near margin.

Margin: Marginal setae with well-developed basal sockets and usually slightly blunt but rarely with simple, pointed apices; length of each seta 17–79 µm; with 4–12 setae present between anterior and posterior stigmatic clefts. Stigmatic clefts shallow or absent, each with 1–4 (usually 3) stigmatic spines, central spine longest, 50–103 µm long, about three to eight times as long as lateral spines.

**Figure 2.**
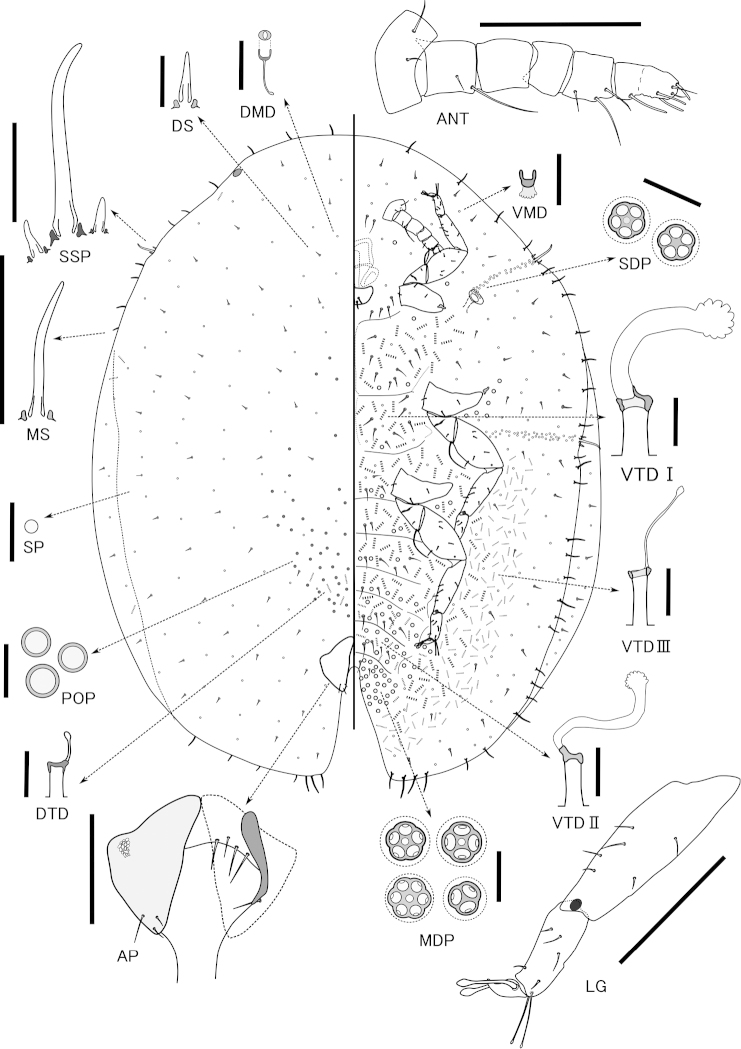
*Pulvinaria caballeroramosae* Tanaka & Kondo, sp. n., adult female. **ANT** antenna **AP** anal plates **DMD** dorsal microduct **DS** dorsal seta **LG** leg **MS** marginal seta **MP** multilocular pores **POP** preopercular pores **SP** simple pore **SDP** spiracular pores **SSP** stigmatic spines **VMD** ventral microduct **VTD** ventral tubular ducts of types I–III. Scales: 200 µm for **ANT**, **LG**; 100 µm for **AP**; 50 µm for **MS**, **SSP**; 10 µm for others.

Venter: Derm membranous. Multilocular pores each 5–9 µm wide, with 3–8 loculi (mainly 5), present around genital opening, on mediolateral areas of all abdominal segments, mesothorax, metathorax and head; a small group also present lateral to each coxa. Spiracular pores each 4.0–7.0 µm wide, with 3–6 loculi (mainly 5), present in rather narrow bands 1–5 pores wide between margin and each spiracle; anterior bands each with 25–47 pores, posterior bands each with 32–49 pores. Ventral microducts scattered evenly throughout, each about 2.0–3.0 µm wide. Preantennal pore not detected. Ventral tubular ducts of three types: type I with large outer ductule, flower-shaped well-developed terminal gland and stout inner ductule, present in medial area of thorax, the anterior two to four abdominal segments, and in inner submarginal band from area posterior to vulvar region near anal folds forwards to area just posterior to metathoracic spiracular pore band; type II tubular ducts each with rather small outer ductule, narrower inner ductule, shallow cup-shaped invagination and well-developed terminal gland, occurring in medial area of posterior abdominal segments; and type III ducts similar to type II, but with a short, filamentous inner ductule and very small terminal gland, present in submarginal band from area posterior to vulvar region near anal folds forwards to area posterior to metathoracic spiracular pore band, intermixed with type I ducts in inner submarginal area. Ventral tubular ducts of all types absent marginally and submarginally from head to anterior thoracic segments and from the outer submarginal to marginal areas of posterior thorax and abdomen. Ventral submarginal setae short and fine, distributed evenly; other ventral setae relatively long and present in medial area of thorax, between antennae and in transverse rows of abdominal segments. Spiracles normal, rather large; width of each peritreme: anterior 90–117 µm, posterior 103–132 µm. Legs well developed and stout, each with a tibio-tarsal articulation and an articulatory sclerosis; claws without denticles; both claw digitules rather broad and slightly shorter than thin tarsal digitules, as shown in Figure 2. Hind trochanter + femur 390–483 µm long, hind tibia 256–325 µm long, and hind tarsus 132–177 µm long. Antennae rather reduced, total length 302–404 µm; each with 5–7 segments, usually 6 or 7. Labium 110–170 µm wide.

#### Etymology.

The species is named after Dr. Andrea Amalia Ramos Portilla and Mr. Alejandro Caballero who originally discovered this soft scale species on the streets of Bogota, Colombia.

#### Biology.

The insects were found on the trunk, branches and twigs of the host. Adult males and puparia were commonly intermixed with the females (Figure 1A). *Pulvinaria caballeroramosae* sp. n. is commonly found in large numbers on *Ficus soatensis* (Figure 1A, B), a common street tree in Bogota, often causing dieback of twigs and branches and in severe cases, dieback of the entire tree. The females produce long ovisacs that are conspicuous on the infested twigs and branches (Figure 1C). No natural enemies, parasitoids or predators of *Pulvinaria caballeroramosae* sp. n. were observed in the present study.

**Host plant.** Moraceae: *Ficus soatensis*.

## Discussion

This species is considered to be close to *Pulvinaria drymiswinteri* Kondo & Gullan based on the distribution pattern of the ventral tubular ducts, tendency for reduction of the antennae and by the way it produces its ovisac, which is strongly adhered to the surface with the eggs exposed and visible through the fibrous ovisac. However, *Pulvinaria caballeroramosae* is easily distinguishable from *Pulvinaria drymiswinteri* by the following combination of features (character states of *Pulvinaria drymiswinteri* in parenthesis): (1) dorsal tubular ducts present (absent); (2), dorsal microducts present (absent); (3) small reticulations on anal plates present (absent), (4) band of preopercular pores broadening anteriorly (not broadening anteriorly, present in a narrow band); and (5) multilocular pores mainly each with five loculi (multilocular pores mainly each with 5–8 loculi).

In the Neotropical region, 27 species of *Pulvinaria* have been recorded (Ben-Dov et al. 2014) of which five are considered to be invasive species in South America (Kondo and Gullan 2010). *Pulvinaria caballeroramosae* sp. n. is considered an urban pest in Bogota, Colombia, because of the damage it causes to *Ficus soatensis* street trees. *Pulvinaria caballeroramosae* sp. n. appears to be an endemic species since it has only been found on a native host, *Ficus soatensis* (Moraceae) in Bogota, Colombia and has not been reported from elsewhere. Furthermore, the second author also examined other fig species while collecting *Pulvinaria caballeroramosae* sp. n., i.e., a less frequent street tree, *Ficus elastica* Roxb. ex Hornem. and *Ficus benjamina* L. (a common ornamental). These *Ficus* spp. were not infested by *Pulvinaria caballeroramosae* despite being in the proximity of infested trees, suggesting that this new *Pulvinaria* species is monophagous. However, further studies are needed in order to determine the host range of this new species of *Pulvinaria*.

Elucidating the taxonomic position of *Pulvinaria caballeroramosae* sp. n. was out of the scope of our study. A comprehensive phylogenetic analysis of the genus *Pulvinaria* of the Neotropical region is needed, using morphological and molecular data, and characters from other instars and males.

## Supplementary Material

XML Treatment for Pulvinaria

XML Treatment for Pulvinaria caballeroramosae
